# Food Palatability Directs Our Eyes Across Contexts

**DOI:** 10.3389/fpsyg.2021.664893

**Published:** 2021-05-31

**Authors:** Yu Liu, Anne Roefs, Chantal Nederkoorn

**Affiliations:** Department of Clinical Psychological Science, Faculty of Psychology and Neuroscience, Maastricht University, Maastricht, Netherlands

**Keywords:** attentional bias, dynamics, context, priming, restrained eating

## Abstract

It is often believed that attentional bias (AB) for food is a stable trait of certain groups, like restrained eaters. However, empirical evidence from this domain is inconsistent. High-calorie foods are double-faceted, as they are both a source of reward and of weight/health concern. Their meaning might depend on the food-related context (i.e., focus on health or on enjoyment), which in turn could affect AB for food. This study primed 85 females with hedonic, healthy, and neutral contexts successively and examined whether food-related context affected AB for food and if effects were moderated by dietary restraint. Both the mean tendencies of AB for food and variability of AB for food were assessed in a food dot-probe task with a recording of both reaction times and eye movements. Contrary to our hypotheses, AB for food was not significantly affected by either context or the interaction between context and dietary restraint. Instead, liking of the presented food stimuli was related to longer initial fixations and longer dwell time on the food stimuli. In addition, in line with prior research, body mass index (BMI) was correlated with variability of AB for food instead of mean AB for food. In conclusion, this study did not find any support that AB for food is dependent on food-related context, but interestingly, reaction time-based variability of AB for food seems to relate to BMI, and eye movement-based mean AB seems to relate to appetitive motivation.

## Introduction

In general, people are naturally attracted by high-calorie foods (e.g., McSorley et al., 2017). In the Western food-rich environment, the abundant presence of high-calorie palatable foods represents salient cues that can induce food craving (Hill and Peters, 1998), subsequent food intake, and ultimately weight gain (Boswell and Kober, 2016). However, in daily life, there are also moments that weight control thoughts or weight/health-related cues can lead to food avoidance. High-calorie foods are frequently craved but are also often a source of worry and weight concern. This is also referred to as the double-facetted nature of food (Roefs et al., 2018), in other words, a conflict between food enjoyment and weight concern. The current study investigates if inducing a hedonic vs. a health context affects attentional bias (AB) for food and if this effect is moderated by dietary restraint.

AB refers to an enhanced attention to salient or relevant stimuli (Drobes et al., 2019). In previous studies, AB for food was mostly considered as a trait-like characteristic of both restrained eaters (REs) and people with overweight/obese. This popular belief is based on the incentive salience model: a reward stimulus, like palatable food, can lead to a craving for it, which reflects on a biased attention to the rewarding food and such attention-grabbing should be especially true for REs and people with overweight/obese (e.g., Berridge, 2009; Nijs and Franken, 2012). However, the empirical evidence for food-related AB in REs and people with overweight/obesity is inconsistent, which has been repeatedly revealed by reviews and meta-analyses (e.g., Doolan et al., 2015; Roefs et al., 2015; Werthmann et al., 2015; Hagan et al., 2020; Hardman et al., 2020). Briefly, some studies indeed found that people high in body mass index (BMI) or dietary restraint biased their attention more to food stimuli than the control group (e.g., Meule et al., 2012; Kemps et al., 2014; Hume et al., 2015), while other studies found that people high in BMI or dietary restraint showed equal (e.g., Werthmann et al., 2013; Doolan et al., 2014; Hodge et al., 2020) or even less (e.g., Nathan et al., 2012; Fang et al., 2019) attention to food cues than their counterparts. Interestingly, some studies even revealed an attention approach-avoidance pattern in people high in BMI and dietary restraint (e.g., Hollitt et al., 2010; Werthmann et al., 2011). This inconsistency might partly be explained by the fact that diverse measurements were used to capture individuals’ AB for food (e.g., different paradigms and materials used to assess AB for food). It could also be due to some studies being underpowered, which possibly could have led to biased results. However, we believe that AB for food is not a stable trait-like feature in people with overweight/obesity and REs.

Apart from the inconsistent empirical evidence, linking enhanced AB for food to people high in BMI or dietary restraint also conveys two incorrect beliefs: (1) food is only a source of reward; (2) attention is stable over time, either towards or away from food. As we stated before, palatable foods can be a source of both reward and weight gain/health concern (Roefs et al., 2018). Relevant for the second incorrect belief is that attention is also a reflection of the current motivation (top-down; Connor et al., 2004). Therefore, theoretically, whether an individual focuses on the enjoyment facet or the health facet of food could direct attention towards or away from food, which might have contributed to the inconsistent results across studies. Previous studies indeed provided evidence that it is possible to influence individuals’ AB for high-calorie foods and subsequent food intake by manipulating the context. For example, in an online supermarket eye-tracking study, priming health/diet cues (recipe banners containing health and dieting words were presented during food choice) increased low-calorie food choices, decreased high-calorie food choices, and increased total dwell times on low-calorie products (van der Laan et al., 2017). Papies and Hamstra (2010) found that priming dieting cues (a weekly recipe that was “good for a slim figure” and low in calories was attached to the door of the butcher’s store) decreased food consumption in REs, but not in unrestrained in unrestrained eaters (uREs). In line with this, a dieting context (participants were asked to choose a 1-day menu from a healthy menu card to their best friend who wants to lose weight) decreased mean AB scores for high-calorie food only in participants with higher dietary restraint scores (Werthmann et al., 2016). All in all, evidence supports the idea that individuals’ AB for food is not stable and is influenced by food-related contexts, especially in high REs.

In the tasks used to measure AB, an aggregated mean AB score has been widely adopted to characterize AB for food, which reflects an overall, stable tendency of AB during the task. However, this mean AB score does not do justice to the potentially dynamic nature of AB for food, as attention to food might alternate between approach and avoidance, even within one study within one participant. Interestingly, another method of computing AB was introduced, trial-level-bias scores (TL-BS; Zvielli et al., 2015), which specifically acknowledges that AB might not be a fixed characteristic but may instead fluctuate over the course of an experiment. This method focuses on the degree of fluctuation in AB and how this fluctuation is related to certain traits. TL-BS has shown added value in several domains. That is, studies revealed that TL-BS variability, one of the parameters to measure the variability of AB for critical stimuli based on the TL-BS, could better predict BMI, depression, and spider phobia than corresponding mean AB scores (Zvielli et al., 2015, 2016; Liu et al., 2019a,b). Specifically, for AB for food, it has been shown that people with a higher BMI do not have significantly more AB for food than healthy-weight people but are characterized by more TL-BS variability for food (Liu et al., 2019a,b).

Altogether, both empirical and theoretical evidences support that individuals’ AB for food is not a stable trait but fluctuates over time. Attention fluctuations might reflect the momentary inner conflict between food enjoyment and weight/health concern when confronted with palatable food, which possibly can be shaped by manipulating food-related contexts. Moreover, individuals with higher dietary restraint are more sensitive to both food-related reward and punishment (Ahern et al., 2010), and as we mentioned before, it was found that the influence of context on food intake and AB for food only happened in participants high in dietary restraint (Papies and Hamstra, 2010; Werthmann et al., 2016). Therefore, it might be that high REs who frequently experience conflicts between food enjoyment and weight concern (Stroebe et al., 2013) have more fluctuations in AB for food when in a “neutral state,” reflecting what might typically occur in daily life, fluctuating between craving and weight concern when confronted with palatable food. These fluctuations might reduce when the context strongly emphasizes either enjoyment or health. Specifically, people may have more and more consistent AB towards high-calorie food when in a hedonic context and more and more consistent AB away from high-calorie food when in a health context. Relevant to mention here is that the mindset or context was not manipulated in previous studies that used TL-BS AB scores. It would therefore be valuable to see how the food-related contexts influence the variability of AB for food.

The current study examined the effect of context priming on both the average AB for food and the variability of AB for food. Context (hedonic, health, and neutral) was manipulated in a within-subject design. It was hypothesized that (1) in the hedonic context as compared with the health context, participants would show a larger mean AB towards food and have longer first fixations and dwell time on food stimuli, whereas the neutral context was expected to fall in between the hedonic and health contexts; (2) compared with the neutral context, participants would show fewer reaction time (RT)-based and eye movement (EM)-based fluctuations on food in both the hedonic and health contexts; (3) contexts would affect both the average and the fluctuations of AB for food more for participants scoring higher on dietary restraint.

## Materials and Methods

The current study was approved by the Ethical Committee of the Faculty of Psychology and Neuroscience, Maastricht University, and all participants signed the informed consent, in which they were informed about the procedure, storage of data, and their right to withdraw from participation without any consequence. This study was pre-registered at AsPredicted.^1^

### Participants

A total of 91 female participants were recruited *via* posters on the university campus (Maastricht University, the Netherlands) or on the Facebook, or *via* an online recruitment system. To disguise the true purpose of this study and avoid influencing participants’ behavior, it was informed on the poster that this study aims to investigate “attention patterns for different objects.” People who signed up for the study received a screening questionnaire with questions on sex, age, and vision. Females with a normal or corrected-to-normal vision and above 16 years old were invited to participate. Because food pictures depicting meat were included in the study, after the participant finished the experiment, she was asked whether she adheres to a vegetarian or vegan diet. Six participants were excluded from analyses because of either a vegetarian or vegan diet. So 85 participants were included in the analyses. Participants’ characteristics can be found in Table 1. The sample size is adequate to detect a medium effect size for the main aim of the study, which is to test the effect of priming condition (within-subject) on all measures of AB scores; thus, repeated-measures MANOVAs were conducted. When using repeated-measures MANOVAs (number of groups = 1, number of measurements = 3, medium effect size *f* = 0.25, alpha = 0.05, power = 0.95), the estimated required sample size was 45.

**Table 1:** Participant characteristics.

Variables	*M*	*SD*	*Range*
RS score	13.80	5.20	1.00–28.00
Age	21.48	2.96	17.00–31.00
BMI	22.02	3.01	16.81–31.25
Hunger	33.40	23.74	0.00–80.00
Food liking	57.12	19.91	6.50–92.50

RS, restraint scale (Herman and Polivy, 1980); BMI, body mass index; hunger, hunger level of participants (0–100 VAS); food liking, average food liking score for the high-calorie food stimuli presented in the food-dot probe task (0–100 VAS).

### Measurements

#### Dietary Restraint

The Restraint Scale (RS, Herman and Polivy, 1980) was used to measure restrained eating. The RS is an 11-item self-report scale that is used to assess chronic dieting. In this study, Cronbach’s alpha was 0.76.

#### Hunger Level

Participants’ hunger level was measured on an online 100-mm visual analog scale (VAS) by asking “How hungry are you right now?,” with 0 indicating absolute lack of hunger and 100 indicating extreme hunger.

#### Liking of the Food Images

Liking of the high-calorie food images that were presented in the food-dot probe task (20 images) was measured on an online 100-ms VAS, with 0 indicating a lack of liking and 100 indicating extreme liking.

#### Food Dot-Probe Task

AB for high-calorie foods was measured using the food dot-probe task with a recording of both RTs and EMs. The task was presented using Experiment Builder (SR Research, Ontario, Canada).

##### Trial Procedure

The priming context was manipulated in a blocked fashion, and in each block, one type of priming picture (hedonic or health or neutral) was presented in each trial. Each trial began with a central fixation dot, which disappeared directly after participants fixated on it. Subsequently, a prime image was presented for 1,000 ms. After presentation of another central fixation dot (500 ms), two images were simultaneously presented side by side for 2,000 ms. Next, one of the images was replaced by the probe (*), which randomly and equally often appeared on the left or right side of the screen. The probe was presented until the participant’s response or for a maximum of 2,000 ms. Participants were instructed to focus on the central fixation and to respond to the probe as quickly as possible.

##### Trial Types

Three different types of trials were included: food-incongruent trials (ITs), food-congruent trials (CTs), and neutral-neutral trials (filler trials). On the ITs and CTs, a high-calorie food and a musical instrument picture were presented, whereas on the filler trials, two neutral nonfood pictures (e.g., office supplies), were presented. During ITs, the probe appeared in the location of the musical instrument picture, whereas during CTs, the probe appeared in the location of the food picture. In the filler trials, the probe appeared randomly and equally often on the left and right sides.

##### Block Types

The task included one practice block, two buffer blocks, and three priming blocks. The three different trial types were evenly and randomly distributed across the priming blocks (40 CTs, 40 ITs, and 40 filler trials for each priming block). Buffer blocks (40 filler trials for each buffer block) only included filler trials and served to neutralize the participants’ mindset. Each priming block included 20 different prime pictures, 10 different food-musical instrument pairs, and 10 different neutral-neutral pairs. Each buffer block included five different neutral prime pictures and 10 different neutral-neutral pairs. The buffer blocks were presented between two priming blocks to reduce the interference from the prime pictures of the previous block. The order of the priming blocks was counterbalanced across participants.

##### Stimuli in the Food Dot-Probe Task

Most of the palatable food stimuli and musical instrument stimuli in the current study were from Werthmann et al. (2011). All image pairs were subjectively matched as closely as possible with regard to the shape, color, brightness, and size of the depicted object. Each picture appeared equally often on the left and right sides of the screen. All the food stimuli were rated by participants in the final online questionnaire according to how much they like the food.

##### Prime Pictures

Ninety candidate prime pictures, with 30 depicting the eating enjoyment context (e.g., wedding), 30 depicting a health-related context (e.g., sports), and 30 depicting a food-unrelated context (e.g., street view), were rated on how much food indulgence and how many healthy food choices they elicited on a 100-mm VAS by asking “How much would you like to indulge in tasty food after viewing the above picture?” and “How inclined are you to choose healthy food after viewing the above picture?,” with 0 reflecting “not at all” and 100 reflecting “very much.” Ratings were provided by 37 women (*M*_age_ = 21, *SD*_age_ = 3.20; no participants of the current study). The 20 eating enjoyment-related pictures with the highest ratings on food indulgence were selected as the hedonic primes; the 20 health-related pictures with the highest ratings on healthy food choices were selected as the health primes; the 20 food-unrelated pictures, with lower ratings on both food indulgence [hedonic primes vs. neutral primes: *t*(19) = 41.26, *p* < 0.001] and healthy food choices [healthy primes vs. neutral primes: *t*(19) = 21.49, *p* < 0.001], were selected as the neutral primes. The prime pictures in the buffer blocks were landscape pictures. For the average priming picture rating scores per priming condition, see Table 2.

**Table 2:** Priming pictures rating scores per priming condition.

	Hedonic primes		Health primes		Neutral primes	
Variables	*M*	*SD*	*M*	*SD*	*M*	*SD*
Indulgence	64.24	2.32	36.05	2.60	37.59	2.17
Healthy food choices	39.58	6.15	60.33	3.21	36.57	4.11

#### Eye-Movement Measurements

Participant’s EM data were collected *via* a desktop mounted EyeLink 1000 system. All stimuli were presented on a 24-inch computer screen at a viewing distance of about 57 cm. With the use of DataViewer software (SR Research, Canada), saccades and fixations were extracted. The display screen was divided into three interest areas: the middle section (represented the location of the fixation cross) and the left and right sections (represented the location of the stimuli). The width of the middle-interest area was decided by a given visual angle: 2 horizontal degrees (Amir et al., 2016). Fixations located in the middle-interest area and fixation durations below 60 ms were discarded (Amir et al., 2016).

### Manipulation Check

The manipulation check was conducted at the end of the experiment, to test whether the three types of prime pictures influenced participants’ desire to indulge in palatable foods differently. All prime pictures used in the task were successively and randomly displayed on the screen in three blocks with the same category of prime pictures in one block. Each picture was presented for 1,000 ms (same display time as in the food dot-probe task). After viewing one block, participants were asked to indicate how much they would like to indulge in tasty food right now on a 100-mm VAS. The three blocks were presented in random order.

### Procedure

All participants were tested individually in a laboratory at Maastricht University after 10 a.m. The order of the priming context was counterbalanced across participants. First, the participant’s hunger level was measured on a 100-mm VAS together with several filler questions^2^ *via* a Qualtrics online survey. Then after a 9-point calibration (calibration for proper gaze recording by the system) with subsequent validation (validation for gaze position accuracy achieved by the current calibration) procedure, the food dot-probe task was administered. Next, the manipulation check, the RS, liking of the food pictures, and self-reported height and weight were assessed in another Qualtrics online survey. Finally, we explained the real purpose of the current study to the participant, and the participant was thanked for her participation and received a small reward (either 1.5-h course credits or 10 euros in gift voucher). The whole procedure lasted about 1.5 h.

### Data Reduction and Analysis

All AB scores were computed separately for each priming condition for each participant.

#### Reaction Time-Based Attentional Bias for Food

Mean AB for food was calculated by subtracting the mean RTs of CTs from the mean RTs of ITs. So a positive value reflects an AB towards food, and a negative value an AB away from food. To obtain sequential TL-BSs, first, each CT was paired with an IT that was as close as possible in time and no further than five trials away. Next, the CT in each pair was subtracted from the IT in that pair. TL-BS variability for food was computed using the sum of absolute distances between sequential TL-BSs divided by the total number of TL-BSs (Zvielli et al., 2015).

#### Eye Movement-Based Attentional Bias for Food

Based on EM data, two average AB for food scores were computed: mean initial fixation duration bias on food and mean dwell time bias on food. The initial fixation duration represents the duration of the first fixation that remains on one of the picture stimuli. Initial fixation durations were firstly averaged across trials per participant, separately for the two categories (food/nonfood). Then, per participant, the mean initial fixation duration bias on food was calculated by subtracting the averaged initial fixation duration in the interest area containing a musical instrument stimulus from the averaged initial fixation duration in the interest area containing a food stimulus.

The dwell time is the total time that a gaze remained at each stimulus during the 2,000-ms presentation time. Mean dwell time bias was calculated by subtracting the mean dwell time in the interest area with a musical instrument stimulus from the mean dwell time in the interest area with a food stimulus. So positive values reflect more attention for food than for musical instruments. The EM-based dynamic changes of AB for food were operationalized as the standard deviation (*SD*) of the initial fixation duration bias on food, the *SD* of dwell time bias on food, and the number of switches between the food and nonfood stimuli within each trial.

#### Data Reduction

Firstly, buffer blocks were excluded from analyses, and then trials were excluded from analyses if they contained error responses, were faster than 200 ms, and slower than 2,000 ms, and after that if they deviated more or less than 3 *SD*s from each participant’s mean RT (Werthmann et al., 2011; 2.20% of the RT data were excluded). In addition, it was checked if participants moved their eyes on a sufficient proportion (50%) of trials (Bradley et al., 2000), and this led to no participant exclusion. Then both RT-based and EM-based AB scores per priming context were calculated separately, after that under each priming context, it was checked whether any AB score deviated more or less than three *SD*s from the respective mean of the whole sample (outliers). Finally, 29 outlier AB scores (the percentage of these outliers was 1.62%) were replaced by the respective nearest score of the whole sample (Wilcox, 2001).^3^

#### Analysis Plan

First, the manipulation check regarding a priming context was conducted by comparing the scores on the manipulation question between a hedonic/health context and a neutral context in a repeated-measures ANOVA. Then, two repeated-measures MANOVAs were conducted to test the effects of different priming contexts and the interaction between priming contexts and dietary restraint (mean-centered) on both AB fluctuation scores (TL-BS variability, *SD* of initial fixation duration bias, *SD* of dwell time bias, and number of switches between the food and nonfood stimuli) and mean AB scores (mean AB scores, mean initial fixation duration bias, and mean dwell time bias). According to previous studies (e.g., Tapper et al., 2010; Hardman et al., 2020), BMI, hunger level, and food liking scores might influence AB scores. Last, correlations between AB fluctuation scores/mean AB scores and these variables were conducted as exploratory analyses. Because of the large number of statistical tests, alphas were adjusted using the Bonferroni method.

## Results

### Manipulation Check

Data were analyzed in a repeated-measures ANOVA with priming condition as the factor and scores on the manipulation check question “how much would you like to indulge in tasty food after viewing the above video” as the dependent variable. Scores on the manipulation check question differed significantly between priming conditions, *F*(2, 168) = 83.50, *p* < 0.001. *Post hoc* tests using the Bonferroni correction (adjusted alpha = 0.025) revealed that ratings were higher for the hedonic condition, *M* = 63.88, *SD* = 2.80, than for both the health, *M* = 36.44, *SD* = 2.71, and the neutral condition, *M* = 35.04, *SD* = 2.84; *ps* < 0.001. The health condition did not differ significantly from the neutral condition, *p* = 1.00. So the hedonic context was successfully induced, but the health context was not.

### Average Performance on Tasks

Participants’ average performance on the task is displayed in Table 3.

**Table 3:** Measures of AB per priming condition.

Variables	Hedonic priming		Healthy priming		Neutral priming	
	*M*	*SD*	*M*	*SD*	*M*	*SD*
TL-BS variability	132.15	49.65	128.77	46.88	136.74	55.97
Mean AB	9.64	25.75	7.83	24.54	8.83	25.65
Mean initial fixation duration bias	39.08	73.51	35.98	67.11	46.39	73.26
Mean dwell time bias	48.80	295.95	15.56	242.53	50.89	211.06
*SD* of initial fixation duration bias	359.02	152.65	375.95	173.08	376.39	186.10
*SD* of dwell time bias	894.42	345.52	911.58	373.72	894.07	363.02
Number of switches	1.84	0.54	1.80	0.66	1.83	0.59

TL-BS, trial-level bias scores; AB, attentional bias; SD, standard deviation.

### Effects of Priming Condition and Restrained Eating on Attentional Bias for Food

#### Mean Attentional Bias for Food

The results from MANOVA showed that there was no statistically significant main effect of priming contexts, *F*(6, 78) = 0.81, *p* = 0.57, Wilks’ *Λ* = 0.94, partial *η*^2^ = 0.06, on the combined dependent variables, mean AB scores. The interaction effect between priming contexts and dietary restraint on the combined dependent variables was also not statistically significant, *F*(6, 78) = 1.87, *p* = 0.10, Wilks’ *Λ* = 0.87, partial *η*^2^ = 0.13.

#### Fluctuations in Attentional Bias for Food

There was no statistically significant difference between the priming contexts on the combined dependent variables, AB fluctuation scores, *F*(8, 76) = 1.20, *p* = 0.31, Wilks’ *Λ* = 0.89, partial *η*^2^ = 0.11. The interaction effect between priming contexts and dietary restraint on the combined dependent variables was also not statistically significant, *F*(8, 76) = 0.73, *p* = 0.66, Wilks’ *Λ* = 0.93, partial *η*^2^ = 0.07.

### Correlational Analyses

#### Mean Attentional Bias for Food

The results of the correlations (adjusted alpha = 0.0167) showed that food liking was positively related to mean initial fixation duration bias and mean dwell time bias. Apart from that, BMI, hunger level, and food liking were not related to any other mean measures of AB scores; see Table 4.

**Table 4:** Correlations between BMI, hunger level, food liking, and mean attentional bias indexes.

*N* = 85	1	2	3	4	5	6
1. Mean AB	1	0.35^*^	0.50^*^	0.001	−0.03	0.21
2. Mean initial fixation duration bias		1	0.85^*^	−0.0003	0.25	0.35^*^
3. Mean dwell time bias			1	0.01	0.19	0.46^*^
4. BMI				1	−0.08	−0.23
5. Hunger					1	0.29^*^
6. Food liking						1

AB, attentional bias; BMI, body mass index; hunger, hunger level of participants (0–100 VAS); food liking, average food liking score for the high-calorie food stimuli presented in the food-dot probe task (0–100 VAS).

* *p* < 0.0167.

#### Fluctuations in Attentional Bias for Food

As for the AB fluctuation scores, only BMI was significantly related to the TL-BS variability: participants with a higher BMI showed more variability in AB for food (adjusted alpha = 0.0125). For more information, see Table 5.

**Table 5:** Correlations between BMI, hunger level, food liking, and attentional bias fluctuation indexes.

*N* = 85	1	2	3	4	5	6	7
1. TL-BS variability	1	−0.12	−0.12	0.02	0.27^*^	−0.0002	0.14
2. *SD* of initial fixation duration bias		1	0.66^*^	−0.57^*^	0.001	0.14	0.13
3. *SD* of dwell time bias			1	−0.65^*^	−0.06	0.05	0.10
4. Number of switches				1	0.07	−0.11	−0.17
5. BMI					1	−0.08	−0.23
6. Hunger						1	0.29^*^
7. Food liking							1

TL-BS, trial-level bias scores; SD, standard deviation; BMI, body mass index; hunger, hunger level of participants (0–100 VAS); food liking, average food liking score for the high-calorie food stimuli presented in the food-dot probe task (0–100 VAS).

* *p* < 0.0125.

## Discussion

This study examined whether hedonic and health priming conditions influence AB for food and how this effect would be moderated by dietary restraint. Both RT-based and EM-based mean tendencies and variability of AB for high-calorie foods were measured. Unexpectedly, it was found that both the priming condition and the interaction between the priming condition and dietary restraint did not significantly affect AB for food. However, BMI was positively related to TL-BS variability, which is in line with previous studies (Liu et al., 2019a,b). In addition, food liking was positively related to initial fixation duration bias and dwell time bias. So participants who reported higher liking of the presented food stimuli looked at the food stimuli longer.

The results of the manipulation check demonstrated that after experiencing hedonic priming pictures, participants reported that they wanted to indulge in high-calorie foods more as compared with both the neutral and health priming contexts, which means the hedonic priming context indeed induced a hedonic goal. However, the priming manipulation was not entirely successful, as there was no significant difference between the health and neutral priming contexts. Thus, it can be concluded that even though the hedonic priming was successful, it did not translate to effects on AB for food. It contradicts a previous study (Werthmann et al., 2016), which found that a health mindset as compared with a palatability mindset decreased RT-based AB for high-calorie foods in participants with higher dietary restraint. However, it should be noted that in Werthmann et al. (2016), the two mindset conditions (health vs. hedonic) only differed significantly on the rated importance of health, but not on the rated importance of palatability, and the design did not include a neutral mindset condition. So less AB for high-calorie foods in a health mindset than in a palatability mindset should likely be attributed to an increased importance of health in the health mindset condition. In addition, to the best of our knowledge, a previous study only found a significant influence of context on AB for food primed participants with a dieting-related context instead of a hedonic context (van der Laan et al., 2017). So maybe health/weight concerns more easily reduce AB for high-calorie foods than that a hedonic focus increases AB for food. Therefore, the unsuccessful manipulation in the health condition in our study might explain why our results are not in line with previous studies. To induce weight concern, more salient cues should be included in future studies, like the scales and weight/dieting-related information instead of the exercise and healthy food pictures used in the current study. It also suggests that mild health cues in real life might not be enough to influence attention, especially when hedonic cues are presented at the same time. Moreover, the current study used a within-subjects design, whereas previous studies (e.g., Werthmann et al., 2016) used a between-subjects design. So it might be that the buffer blocks were not sufficient to avoid spillover between the different priming conditions. The current study only investigated the effect of context priming on AB for palatable foods. It would be interesting to investigate how different contexts affect AB for healthy food stimuli in future studies.

Dietary restraint and BMI are frequently believed to be related to AB for high-calorie palatable food, and this has been tested in many studies (e.g., Castellanos et al., 2009; Meule et al., 2012). However, the empirical evidence does not support this claim (e.g., Doolan et al., 2015; Roefs et al., 2015; Werthmann et al., 2015; Hagan et al., 2020; Hardman et al., 2020). The current study also did not find any significant relationship between dietary restraint or BMI and mean AB for food, not even in the hedonic priming condition. So the results from the current study question the notion that AB for high-calorie palatable food is a trait-like feature of people with a high BMI or scoring high on dietary restraint.

Interestingly, the current study did show a positive association between BMI and TL-BS variability for food, which is in accordance with previous studies (Liu et al., 2019a,b), which included a range of participants (normal-weight females and children, overweight females, and overweight/obese children). Notably, in one of these studies (Liu et al., 2019b), it was found that this relation between BMI and TL-BS variability does not hold if attention control is high and another study directly found that the relationship between anxiety and the TL-BS variability for anxiety-related stimuli was significantly mediated by attention control (Clarke et al., 2020). Therefore, the variability of AB for food might reflect weaker executive control. In detail, weaker executive control might make people less likely to have a consistent, prolonged attention to food stimuli during the task, therefore causing more variability in AB for food.

The current study also found positive associations between participants’ food liking and EM-based mean AB for food (both mean initial fixation duration bias and mean dwell time bias), which is consistent with previous studies (e.g., Kemps and Tiggemann, 2009). The incentive salience model (Berridge, 2009) proposed that AB for food reflects appetitive motivation, and except for the influence of momentary motivation on AB for food, the relatively stable trait, food liking, should be also closely related to AB for food. However, the average food liking score was relatively low in the current study, although the food stimuli used in the current study included only widely liked food items, like chocolate and crisps. Tailoring food stimuli to the individual participant should be considered in future studies.

Except for the within-subject design and relatively low food liking score, there are several other limitations of the current study that need to be mentioned here. First, the participants included in this study were fairly homogeneous. They are all females, and most of them were students. Future studies are needed to confirm the effect of context priming on AB for food in diverse populations. Second, because BMI is not our main focus, we used self-reported BMI instead of measuring BMI, which could have caused inaccuracy and therefore have affected the results regarding BMI.

## Conclusion

The present study assessed the effect of context on AB for food. The situational context was manipulated by priming participants with hedonic, healthy, and neutral pictures. Contrary to our hypothesis, the current results did not provide evidence on the influence of a hedonic or health-related context on individuals’ AB for food. A positive association between BMI and TL-BS variability for food was found, which replicates results from previous studies (Liu et al., 2019a,b). Food liking was positively related to AB for food, which is in line with the idea that AB for food reflects appetitive motivation (Field et al., 2016). Finally, adding to the inconsistency in the field (e.g., Doolan et al., 2015; Roefs et al., 2015; Werthmann et al., 2015; Hagan et al., 2020; Hardman et al., 2020), this study failed to show any significant relationship between dietary restraint or BMI and average AB for food.

## Data Availability Statement

The original contributions presented in the study are included in the article/Supplementary Material, further inquiries can be directed to the corresponding author.

## Ethics Statement

The studies involving human participants were reviewed and approved by the Ethical Committee of the Faculty of Psychology and Neuroscience, Maastricht University. Written informed consent from the participants’ legal guardian/next of kin was not required to participate in this study in accordance with the national legislation and the institutional requirements.

## Author Contributions

YL, AR, and CN designed the study. YL collected and analyzed the data. YL wrote the manuscript. AR and CN gave feedback on the manuscript. All authors contributed to the article and approved the submitted version.

### Conflict of Interest

The authors declare that the research was conducted in the absence of any commercial or financial relationships that could be construed as a potential conflict of interest.

## Footnotes

^1^https://aspredicted.org/96j53.pdf

^2^Filler questions: How many glasses of alcohol did you drink yesterday? How many cups of coffee did you drink yesterday? How many cigarettes did you smoke yesterday? How many cups of water did you drink yesterday?

^3^The analyses with unchanged values or exclusion of these values showed the same pattern of results and led to the same conclusions.
