# Supplementary material for: Food Palatability Directs Our Eyes Across Contexts
Source: Front Psychol. 2021 May 31;12:664893. doi: 10.3389/fpsyg.2021.664893 (PMC8201788; doi:10.3389/fpsyg.2021.664893)
Supplement: Supplementary file 1 [file Data_Sheet_1.zip › Data/Explanatory memo.docx]

**An explanation of all files and** **documents**

- The file “RawData” included all raw data from this study.
  - The document "RT Raw " is raw data in the food dot-probe task.
  - The document “FixDwell Raw” is raw eye-movements data used to calculate the first fixation duration and dwell time.
  - The document “Switches Raw” is raw eye-movements data used to calculate switches.
  - The document “Surveys” is survey data.
- The file “TL-BS code” included codes (MATLAB) to compute TL-BS variability and mean AB.
- The file “Output” included attentional bias scores after data reductions, the SPSS output of data analyses, and SPSS syntax of data analyses.

**Remark:**

Reaction-time-based attentional bias scores were computed via MATLAB (TL-BS code). Data analyses were conducted via SPSS.
