# Supplementary material for: Food Palatability Directs Our Eyes Across Contexts
Source: Front Psychol. 2021 May 31;12:664893. doi: 10.3389/fpsyg.2021.664893 (PMC8201788; doi:10.3389/fpsyg.2021.664893)
Supplement: Supplementary file 1 [file Data_Sheet_1.zip › Data/TLBS_code/TLBS Instruction.pdf]

Ariel Zvielli & Amit Bernstein
Department of Psychology
University of Haifa
Our Lab: http://psy.haifa.ac.il/~anxiety_research/

# Computation Code for the Analysis of Trial Level Attentional Bias Scores: Description and General Guidelines for Use by Independent Investigators

**DISCLAIMER: (a) This code is provided for use by independent investigators for analysis of *research data exclusively*. (b) The code below is based on ongoing research and development (R&D) and is expected to change and be updated regularly. (d) Use of this code, necessary input entry or revision for the code, appropriateness of the research data for computation of trial level scores and TLBS bias dynamics parameter computations, management of input data for analysis, etc. - are the full responsibility of the independent user.**

**Trial-level bias score analysis: Brief background.** The code below is based on conceptualization and operationalization of attentional bias or dysregulated attentional processing of emotion or motivationally-relevant information as a **dynamic process in time** (see "Temporal Dynamics of Attentional Bias" - Zvielli, Bernstein, & Koster, 2015; see also "Targeting Biased Emotional Attention to Threat as a Dynamic Process in Time: Attention Feedback Awareness and Control Training (A-FACT)" - Zvielli, Amir, Goldstein, & Bernstein, 2016).

**Sharing the code: Our purposes.** We share this code so that fellow researchers who wish to study the temporal dynamics of attentional bias via TL-BS analyses can do so on their own.

**The Code: Its basic functions. The code subtracts reaction time (RT) between matched trials in order to estimate biased attentional allocation at the trial-level.** In general, **the input** for the code is RT data (e.g., Dot-Probe task) and key information about the data structure and each trial (typically created by E-Prime or similar experimental software). In general, **the output** is two-fold: (1) a time series of trial-level estimations of attentional bias per subject; and (2) a set of dynamic features (parameters) of TL-BS (e.g., mean TL-BS towards, TL-BS variability). The code may be adjusted to make similar computations for other tasks (e.g., spatial cueing, visual search).

**Additional Consideration for Users.** Please note that this code represents one of a number of possible approaches for estimating attentional bias or attentional (dys)regulation at the trial-level. This computation approach is the focus of ongoing research and development; accordingly, ongoing changes/updates/revisions of the code are expected. The dynamic features (parameters) of TL-BS scores are only initial candidate parameters of the dynamics; revision of these parameters, introduction of new parameters, etc are expected.

**Methodological attributes important for TLBS computation:** Many attributes of the data must be taken into consideration by investigators when deciding whether this or some permutation of this code is meaningfully applicable to a specific task or dataset. We **do not** detail all of these considerations here. It is up to investigators to have a strong enough understanding of the cognitive-experimental task

Ariel Zvielli & Amit Bernstein
Department of Psychology
University of Haifa
Our Lab: http://psy.haifa.ac.il/~anxiety_research/
methods as well as the computational and conceptual considerations to apply to these methods responsibly. **Some key considerations** include:

(1) Multiple "conditions" (e.g., categories of emotion stimuli; different presentation durations; number of blocks or sessions; or various other types of variations of trial types) are commonly randomly presented (inter-mixed) within task blocks or across task trials. Depending on your conceptual aims, it is typically the case that you will aim to compute TLBS per "condition". In such a case, we recommend preparing the input separately for the trials of a specific condition and running the code separately on each subset of the data). Again, depending on various task parameters (e.g., # of trials, # of conditions, inter-stimulus interval durations, etc.) may or may not be feasible, and may yield more or less robust estimations of trial-level bias in time.

(2) We generally recommend not computing TLBS parameters on less than ~**40 TLBS computations** per person. This is a bar minimum. In that if you wish to assess reliability (split-half) we recommend at least **80 TLBS computations.**

(3) Any of the above mentioned "conditions" may eventually result in not having sufficient number of trials in each condition (e.g., 80 trials of 4 distinct emotional categories would allow 20 or less TLBS estimations per person, which would be insufficient for the computation of meaningful/reliable TL-BS parameters. "Blind" application of TLBS computations (i.e., "lets see what we get approach") to cognitive-experimental task data (e.g., dot probe, spatial cueing, visual search data, etc.) is a bad idea.

**Code Use: Technical Instructions.**

**Input preparation.** Input data needs to be organized in a single "long-file" of trial-level data (i.e., with one row per trial per participant). Rows need to be sorted, first, by subject number, and second, by ORIGINAL trial index reflecting the actual order trials were presented to each participant. Rows (trials) with error responses, or outlier response times (e.g., RT>1500 or RT<200; RT>3 SD from participants mean) need to be removed from the file before TL-BS analysis. Investigators are responsible for screening and cleaning data based on established practices for RT data and the unique features of their task design parameters.

**Input format.** See Table 1. Columns must be sorted as follows (the **actual file must contain numbers only** and no headers; see example input file attached), and saved as Excel (or .CSV file):

**Table 1. Input Data Format**

| Column | 1 | 2 | 3 | 4 | 5 |
|---|---|---|---|---|---|
| Variable | Subject Number | Trial Index | Response time (ms) | Congruency* | Block Number** |
| Possible values | 1-N, integer, > 0 | 1-N<br>integer, > 0 | integer, > 0 | {1,2,3} | 1-N,<br>integer, positive |

Ariel Zvielli & Amit Bernstein
Department of Psychology
University of Haifa
Our Lab: http://psy.haifa.ac.il/~anxiety_research/
***Congruency**: 1 = Incongruent trial , 2 = Congruent trial 3 = Neutral-Neutral trial.

****Block Number**: If participants have a small break within the session, each segment (before and after the break) will get a consequent number (e.g., 1,2…). This is important to ensure that trials will not be matched between blocks/segments if doing so violates assumptions of time continuity between trials. If there was only one block, column #5 should contain '1' throughout.

**Table 2. Input Data Illustration**

| | | | | |
|---|---|---|---|---|
| 101 | 1 | 340 | 1 | 1 |
| 101 | 2 | 565 | 1 | 1 |
| 101 | 3 | 490 | 3 | 2 |
| 101 | 4 | 480 | 2 | 2 |
| 101 | 5 | 210 | 1 | 2 |
| 102 | 1 | 970 | 2 | 1 |
| 102 | 2 | 840 | 2 | 1 |
| 102 | 3 | 510 | 3 | 1 |

**Illustrative Input – Explanatory Note.** Here (Table 2) you can see a section of an input data table. The first participant (identified by the number '101'), has 5 trials (as indicated by 1-5 trial index column 3; for presentation purposes, usually there are many more trials per participant); a short break was taken after the first two trials, which is marked by the different block number (column 5). .

**Output description.** The code produces two output files:

**(1) Trial-Level Output Data.** An Excel file that contains the same table as in the input file, but with the addition of columns specifying TLBS computation per trial (first-level time-series data), in the event that such computations were possible. Table 3 below is an illustration of this output file format.

**Table 3. Output Format – Trial-Level Data**

| Column | 1-5 | 6 | 7 | 8 | 9 |
|---|---|---|---|---|---|
| Variable | [Input as described above] | TLBS (IT-CT)* | TLBS (IT-CT) Matching Distance | TLBS (Neutral Match) ** | TLBS (Neutral Match) Matching Distance |

***IT-CT**: This computation is done be matching and then subtracting congruent from incongruent trials' RT. (e.g., Zvielli, Bernstein & Koster, 2015).
****Neutral Match**: This computation is done be matching and then subtracting congruent or incongruent trials' RT from Neutral trials' RT (e.g., Zvielli, Amir, Goldstein & Bernstein, 2016). Selection of type of computation must be determined a priori based on conceptual aim analysis and various key methodological parameters of the task and data.

**(2) TLBS Parameters Output Data.** An Excel file that contains the TL-BS parameters or features of the bias dynamics (i.e., second-level data/variables). Table 4 below is an illustration of this output file format for a single participant.

Ariel Zvielli & Amit Bernstein
Department of Psychology
University of Haifa
Our Lab: http://psy.haifa.ac.il/~anxiety_research/

**Table 4. Output Format – TLBS Parameters**

| Column | 1 | 2 | 3 | 4 | 5 | 6 |
|---|---|---|---|---|---|---|
| Variable | SubjNum | traditionalBS | MeanTwd | MeanAwy | PeakTwd | PeakAwy |
| Description | Participant's number | Aggregated mean Bias Score (IT-CT) | Mean TLBS >=0 | Mean TLBS <=0 | Peak (maximum) TLBS towards | Peak (minimum) TLBS away |
| Column | 7 | 8 | 9 | 10 | 11 | 12 |
| Variable | Variability | MeanTwdNT | MeanAwyNT | PeakTwdNT | PeakAwyNT | VariabilityNT |
| Description | SD (TLBS) | Mean TLBS (Neutral Match) >=0 | Mean TLBS (Neutral Match) <=0 | Maximum TLBS (Neutral Match) towards | Minimum TLBS (Neutral Match) away | SD(TLBS Neutral Match) |
| Column | 13 | 14 | 15 | 16 | 17 | 18 |
| Variable | NumMatches | NumMatchesNT | MeanDist | MeanDistNT | SDDists | SDDistsNT |
| Description | Number of TLBS values | Number of TLBS values (Neutral Match) | Mean distance between TLBS matches | Mean distance between TLBS matches (Neutral Match) | SD of distances between matches | SD of distances between matches (Neutral Match) |

**MATLAB Instructions for Code Use: Overview.**

1. Place the attached files in a directory that Matlab can access.
2. Edit input/output file names in "MainTLBS.m".
3. Run "MainTLBS.m".
